# Structural and mitochondrial dendritic degenerations in old hypoglossal motor neurons

**DOI:** 10.1007/s11357-025-02075-w

**Published:** 2026-01-02

**Authors:** Trace A. Christensen, Matthew J. Fogarty

**Affiliations:** 1 Department of Laboratory Medicine and Pathology, Mayo Clinic, Rochester, MN 55905 USA; 2 Department of Physiology & Biomedical Engineering, Mayo Clinic, 200 1st St SW, Rochester, MN 55905 USA; 3 Department of Neurology, Mayo Clinic, Rochester, MN 55905 USA

**Keywords:** Dendritic spines, Electron microscopy, Tongue, Sarcopenia, Neurodegeneration

## Abstract

Hypoglossal motor neurons (MNs) within the medullary hypoglossal nucleus innervate the striated muscles of the intrinsic and extrinsic tongue. Dysfunction of the control of the tongue muscles may lead to problems such as dysphagia, dysphonia and the increased risk of aspiration pneumonia in the elderly. In the human and Fischer 344 (F344) rat motor systems, age-related muscle weakness and behavioural dysfunctions are contemporaneous to MN death. In other neurons, dendritic and mitochondrial degenerations are fundamental pathophysiological components preceding neuronal death. We aimed to determine if dendritic, dendritic spine and dendritic mitochondrial pathology were present in old age. We used golgi-cox and serial block-face scanning electron microscopy (SBFSEM) to evaluate dendritic and mitochondrial morphology, respectively in young (6-month) and old (24-month) female and male F344 rats. Dendritic regression and dendritic spine loss occurs in old age, predominantly in larger hypoglossal MNs. In addition, reduced dendritic mitochondrial volume density and mitochondrial fragmentation are apparent in old age. Our results are consistent with established age-related deficits in F344 rats, including tongue muscle sarcopenia, hypoglossal MN loss and dysphagia. Although more work is needed to determine if synaptic and mitochondrial degenerations are causative for age-related neuromotor dysfunctions, our results suggest that strategies to preserve dendrites and mitochondria may be of therapeutic utility.

## Introduction

Elderly humans suffer from aerodigestive deficits such as weakened cough, dysphagia and subsequent aspiration pneumonia [1–3]. Weakened contractility of respiratory striated muscles, particularly the tongue, may contribute to these outcomes in humans [1–6]. Tongue weakness and atrophy (i.e., sarcopenia) is also evident in a variety of rodent models of aging [7–9], including the Fischer 344 (F344) rat model [10], which also exhibits hypoglossal motor neuron (MN) death at old age (24-months-old) [11]. In respiratory-related motor units, fast fatiguable (type FF) motor units (i.e., larger MNs and the type IIx/b muscle fibers they innervate) seem more susceptible to the ravages of age compared to slow (type S) and fast fatigue-resistant (type FR) motor units, comprised of smaller MNs and the type I or IIa fibers they innervate, respectively [10, 12–15].

Although frank weakness is detrimental, altered synchrony between respiratory and aerodigestive behaviours may be a far more pernicious contributor to morbidity/mortality [16–20]. For the tongue, hypoglossal MN dendrites are responsible for integrating the myriad motor and sensory inputs essential for effective aerodigestive behaviours. In other rodent models of conditions where MN death and muscle weakness occurs, such as amyotrophic lateral sclerosis (ALS), dendritic alterations and dendritic spine loss precede MN death and denervation by some time [21]. In F344 rats, other brainstem MNs, dendritic arbours are reduced in late middle-age, prior to behavioural weakness [19]. It is currently unknown whether hypoglossal MNs in old age exhibit altered dendritic arbours and dendritic spines compared to younger animals.

In F344 respiratory striated muscle (diaphragm [DIAm]), we have found that mitochondrial volume density and function are altered in old age [22, 23], consistent with DIAm denervation and sarcopenia [12, 24, 25]. Indeed, the most extensively validated rat and mouse model of ALS is predicated on a SOD1 mitochondrial mutation where fragmentation and structural abnormalities of mitochondria are readily observed in neurons [26]. To date, the structure of mitochondria in aging MNs has been overlooked.

Here, for the first time we present observations of dendritic and dendritic spine degenerations in hypoglossal MNs of 24-month-old F344 rats. These changes disproportionately affected larger hypoglossal MNs. In 24-month-old F344 rats, hypoglossal MN dendrites had lower mitochondrial volume density and a greater amount of mitochondrial fragmentation.

## Materials and methods

### Ethical approval

All procedures were performed in accordance with the American Physiological Society’s Animal Care Guidelines, the National Institutes of Health (NIH) guide for the use and care of laboratory animals and ARRIVE guidelines. Studies were approved by the Institutional Animal Care and Use Committee (IACUC) at Mayo Clinic (Approval A00008010-24).

### Experimental animals and anaesthesia

In the present study, 24 pathogen-free F344 rats (12 females and 12 males) were obtained from the NIA colony and acclimated at least 1 week before terminal experiments were performed on young (mean: 6-months old), and old rats (mean: 24-months old). Age selection was based on survival information (100%, and ~ 50%, respectively) [27]. Animals were maintained two per cage under an alternating 12:12 h light–dark cycle with ad libitum access to food (rat chow) and water. Prior to all experiments, rats were deeply anesthetized with intraperitoneal injection of ketamine (80 mg/kg) and xylazine (20 mg/kg), indicated by the absence of deep pain and palpebral reflexes.

### Golgi-Cox terminal procedures, processing, imaging & analysis

A subset of rats ( *n* = 6 young; *n* = 6 old) underwent Golgi-Cox impregnation to assess dendritic and dendritic spine morphology [28]. Under deep anaesthesia (indicated by the absence of deep pain and palpebral reflexes, spontaneous whisking and licking, achieved via intraperitoneal ketamine [80 mg/kg] and xylazine [20 mg/kg]), rats were exsanguinated transcardially. Following this euthanasia, the fresh (unfixed) brainstem was removed and processed for 16–18 days in impregnation solution (FD Rapid Golgi, FD NeuroTechnologies) [28], changed once after 24 h. Following impregnation, the brainstem was frozen in melting isopentane and prepared for cryosectioning at 180 µm [19, 29]. Transverse-sectioned brainstem slices were left on slides to dry overnight at 24 °C and then developed (FD NeuroTechnologies), dehydrated with ethanol and xylene and coverslipped. The hypoglossal nucleus was imaged under brightfield illumination with a 40X oil objective (1.3 NA, 1600 × 1600 pixel array) using a pseudo-confocal (1.67 µm step size, 200 nm pinhole) method, creating mosaic images of neurons within the region [30] and analysed in 3D using Neurolucida 11 Desktop Edition (MBF Bioscience) in a manner identical to our past MN assessments [19, 29, 31–35].

### Serial block-face scanning electron microscopy (SBFSEM)

Mitochondrial volume density in hypoglossal MN dendrites was assessed in a separate cohort of 12 rats (6 per age; 3 females and 3 males per group) anesthetised via an intraperitoneal injection of ketamine (80 mg/kg) and xylazine (20 mg/kg). While under anaesthesia, the brainstem was freshly dissected and carefully placed in a dissection dish filled with 0.1 M phosphate buffer solution (PBS). Under a dissection microscope, the dorsal medulla was bisected in the sagittal plane, followed by the removal of rostral and caudal regions greater than ~ 3 mm from the obex. The sample was placed in fixative (2% glutaraldehyde and 2% paraformaldehyde in 0.15 M cacodylate buffer, pH7.5), rinsed in cacodylate buffer, then subsequently postfixed and stained using an osmium/thiocarbohydrazide/osmium (OTO) protocol [36]. Semi-thin survey Sects. (200 nm) were used to select areas of the block that contained hypoglossal MNs. These areas were then mounted to aluminum stubs, trimmed to a 0.5 mm^3^ tower, and sputter-coated with gold to mitigate charge while imaging. Serial block-face images were collected at 20 nm × 20 nm × 75 nm (x,y,z) resolution using a VolumeScope2 SEM (Thermo Fisher) [37, 38].

Hypoglossal MN dendrites were consistent with neurons from other brain regions and differentiated from axons by the absence of myelin and from MN somas by the size (< 5 µm in diameter). Within these images, mitochondria were readily identified, characterised by the presence of internal cristae and location within the dendritic cytoplasm. To sample the volume density of mitochondria within these samples, a Cavalieri volume estimation scheme was employed [39]. Within hypoglossal MN dendrites, individual mitochondrion areas were manually traced, and their area, circularity, perimeters, and long- and short-axes recorded. Thereafter, the dendritic boundary was traced and the area determined. At 0.225 µm intervals from this centre, Cavalieri *z*-slices were obtained in both directions. This process was then repeated for each hypoglossal MN dendrite sampled. The mean area density of mitochondria were determined for each Cavalieri *z*-slice, and a mitochondrial volumes and volume density was calculated based on the number of total slices assessed and the distance between each slice (0.225 µm), for each dendrite, in a manner similarly used in spinal MNs [40].

### Statistical analysis

We used Prism 11 for all data analyses (Graphpad, Carlsbad, CA). Each data set was assessed for normality with D’Agostino and Pearson tests. For single comparisons, unpaired Student’s t-test or Mann–Whitney U-tests were used, the latter if data were non-gaussian. Pearson’s coefficients were used to compare XY relationships. For multiple variables, 2-way ANOVAs with Bonferroni post tests were used. All data are reported as the mean ± the standard deviation (SD) of the mean. Statistical significance was established at the *P* < 0.05 level, reported precisely to four decimal places. In an effort to ensure statistical differences were meaningful, reliable and robust [41, 42], the magnitude of the effect was established by using Cohen’s *d*. Experiments and analyses were performed blind to age and sex. Major outcome measures stratified by age and sex are presented in Table 1.

**Table 1:** Sex-stratified major outcome measures

Parameter	6-months-old (*n*)	24-months-old (*n)*	2-Way ANOVA
Dendritic Length (µm)	♀ 2431 ± 715 [43] ♂ 2733 ± 799 [44]	♀ 1981 ± 610 [44] ♂ 1942 ± 665 [44]	Sex: *P* = 0.1539 Age: *P* < 0.0001
Dendritic SA (µm^2^)	♀ 11,212 ± 5067 [43] ♂ 11,506 ± 4487 [44]	♀ 9093 ± 3287 [44] ♂ 8797 ± 3501 [44]	Sex: *P* = 0.9986 Age: *P* < 0.0001
Spine Density (per 100 µm)	♀ 19.8 ± 6.6 [43] ♂ 17.3 ± 5.7 [44]	♀ 9.3 ± 3.2 [44] ♂ 13.7 ± 4.2 [44]	Sex: *P* = 0.1741 Age: *P* < 0.0001
Mitochondrial Volume Density (%)	♀ 13.6 ± 6.2 [45] ♂ 12.3 ± 5.1 [30]	♀ 5.9 ± 3.9 [38] ♂ 5.1 ± 2.7 [36]	Sex: *P* = 0.1697 Age: *P* < 0.0001
Mitochondrial CSA (µm^2^)	♀ 0.49 ± 0.28 [45] ♂ 0.55 ± 0.28 [30]	♀ 0.31 ± 0.18 [38] ♂ 0.24 ± 0.15 [36]	Sex: *P* = 0.9077 Age: *P* < 0.0001
Mitochondrial Form Factor	♀ 1.72 ± 0.52 [45] ♂ 1.95 ± 0.47 [30]	♀ 1.60 ± 0.48 [38] ♂ 1.51 ± 0.35 [36]	Sex: *P* = 0.2608 Age: *P* = 0.0003

All data mean ± standard deviation. 3 female and 3 male rats per experimental age

## Results

### Rat characteristics across the agespan

Bodyweights did not differ with age (F = 0.3; *P* = 06218), however sex did have an effect (F = 23.9; *P* < 0.0001), with females (~ 270 g) ~ 75% the bodyweight of males (~ 360 g).

### Somal size-dependent reduction of hypoglossal MN dendritic arbour length and surface area in old age

**Fig. 1:**
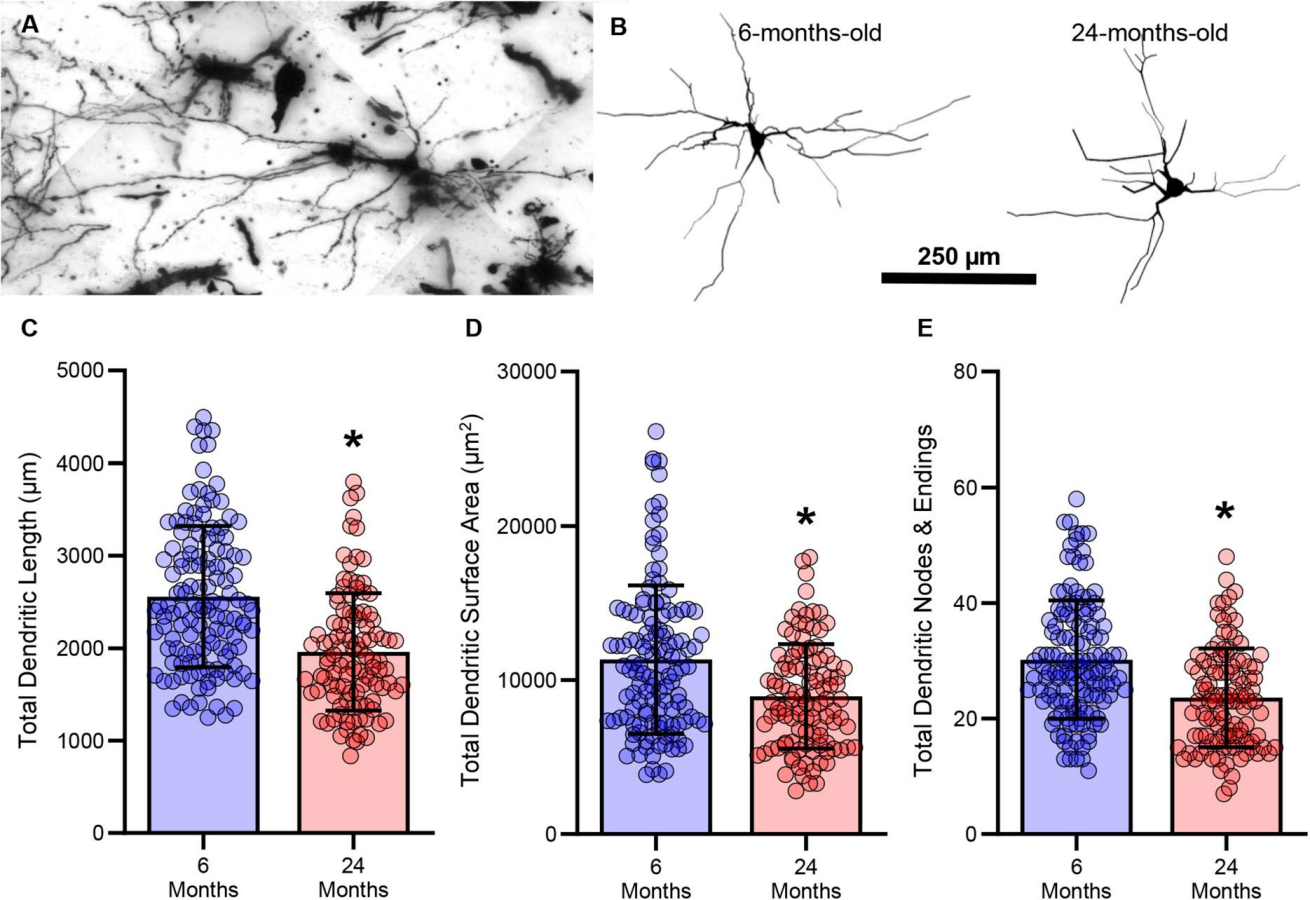
**A **Example projection of golgi-cox impregnated hypoglossal MN image z-stacks. **B **example 3D reconstructions of hypoglossal MNs from 6- and 24-month-old rats. **C **Scatterplot of reduced total hypoglossal MN dendritic length in 24- compared to 6-month-old rats (*P* < 0.0001). **D **Scatterplot of reduced total hypoglossal MN dendritic surface area in 24- compared to 6-month-old rats (*P* = 0.0001). **E **Scatterplot of reduced total hypoglossal MN dendritic nodes and ramifications in 24- compared to 6-month-old rats (*P* = 0.0001). Each dot represents one MN (the *n*), * indicates signiificant difference (i.e., *P* < 0.05)

Here, we assessed the dendritic arbours of hypoglossal MNs from 6- (*n* = 129) and 24-months-old (*n* = 108) in F344 rats (Fig. 1). The general morphological properties of hypoglossal MNs differed with age. The total dendritic arbour length of hypoglossal MNs was reduced by 23% (*P* < 0.0001, Mann–Whitney U-test; large effect size d = 0.85) in 24- (1961 ± 635 µm) compared to 6-month-old (2557 ± 763 µm) F344 rats (Fig. 1C). The total dendritic arbour surface area of hypoglossal MNs was reduced by 21% (*P* = 0.0001, Mann–Whitney U-test; medium effect size d = 0.57) in 24- (8945 ± 3383 µm^2^) compared to 6-month-old (11,335 ± 4817 µm^2^) F344 rats (Fig. 1D). The number of dendritic ramifications of hypoglossal MNs was reduced by 22% (*P* < 0.0001, Student’s unpaired t-test; medium effect size d = 0.70) in 24- (23.6 ± 8.5) compared to 6-month-old (30.2 ± 10.5) F344 rats (Fig. 1E). We did not observe any effects of sex on dendritic length or dendritic surface area (Table 1).

**Fig. 2:**
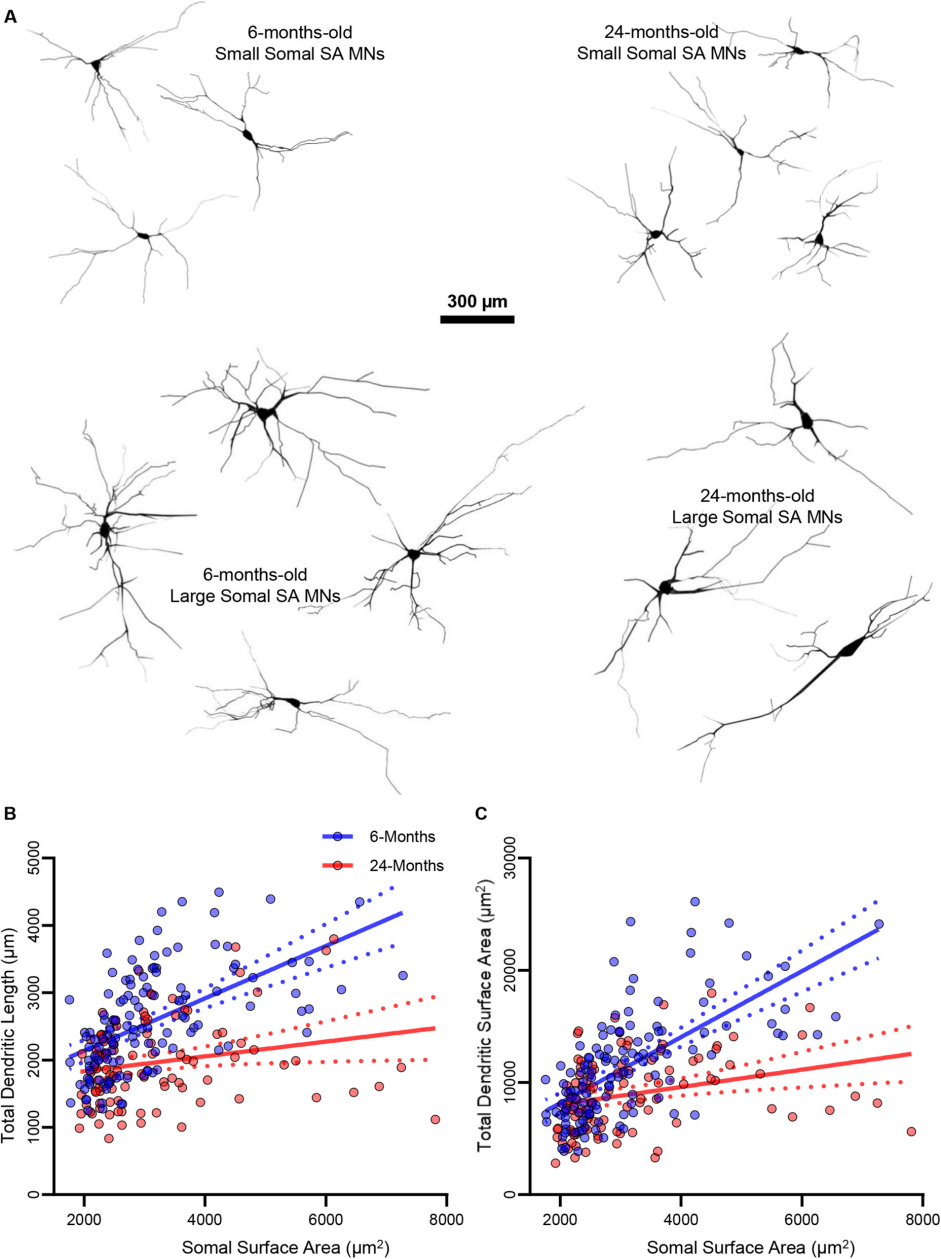
**A **Example 3D reconstructions of hypoglossal MNs with small and large somal surface areas from 6- and 24-month-old rats. **B **XY plot of the positive linear relationship between hypoglossal MN somal surface area and dendritic length in 6- (slope = 0.39, *P* < 0.0001; *r*^2^ = 0.30), but not 24-month-old rats (slope = 0.11, *P* = 0.1100; *r*^2^ = 0.05), with a significant difference between the two co-efficients (*P* = 0.0001). **C **XY plot of the positive linear relationship between hypoglossal MN somal surface area and dendritic surface area in 6- (slope = 2.94, *P* < 0.0001; *r*^2^ = 0.43) and to a lesser extent 24-month-old rats (slope = 0.78, *P* = 0.0035; *r*.^2^ = 0.05), with a significant difference between the two co-efficients (*P* < 0.0001). Each dot represents one MN (the *n*)

When we evaluated dendritic length with regard to somal surface area, 6-month-old hypoglossal MNs exhibited a linear relationship (slope = 0.39, *P* < 0.0001; *r*^2^ = 0.30), while 24-month-old hypoglossal MN dendritic length did not scale as readily with size (slope = 0.11, *P* = 0.1910; *r*^2^ = 0.05; Fig. 2B). The slope of this relationship was reduced by 72% in 24-month-old hypoglossal MNs (*P* = 0.0001; large effect size d = 0.50), indicating larger surface area MNs is where dendritic regression was most apparent (Fig. 2B).

When we evaluated dendritic surface area with regard to somal surface area, 6-month-old hypoglossal MNs exhibited a linear relationship (slope = 2.94, *P* < 0.0001; *r*^2^ = 0.43), while 24-month-old hypoglossal MN dendritic surface area did so to a lesser extent (slope = 0.78, *P* = 0.0035; *r*^2^ = 0.08; Fig. 2C). The slope of this relationship was reduced by 75% in 24-month-old hypoglossal MNs (*P* < 0.0001; medium effect size d = 0.69), indicating larger surface area MNs is where dendritic regression was most apparent (Fig. 2C).

### Dendritic alterations of hypoglossal MNs in aging are specific to distal dendrites

**Fig. 3:**
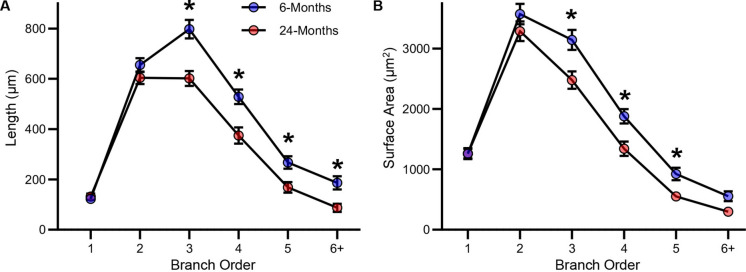
**A **Plot of reduced segmental dendritic length of hypoglossal MNs from the 3rd to the 6th and greater branch orders at 24- compared to 6-months-old (age:* P* < 0.0001, branch order: *P* < 0.0001). * signifies a significant difference (*P* < 0.05) within a branch order using a Bonferroni post-test. **B **Plot of reduced segmental dendritic surface areas of hypoglossal MNs from the 3rd to the 5th branch orders at 24- compared to 6-months-old (age:* P* = 0.0003, branch order: *P* < 0.0001). * signifies a significant difference (*P* < 0.05) within a branch order using a Bonferroni post-test

The distal dendrites are a major site of integrating disparate inputs (particularly excitatory inputs) from a variety of neural regions [46]. Here, we assessed the total dendritic length and surface areas per branch order of hypoglossal MNs from 6- and 24-month-old F344 rats (Fig. 3). The total dendritic segment length per branch order of hypoglossal MNs was dependent on age (F = 40.5, *P* < 0.0001) and branch order (F = 197.7, *P* < 0.0001, 2-Way ANOVA, Fig. 3A). In 24-month-old F344 rats, the total dendritic segment lengths were reduced compared to 6-months-old at the 3rd through the 6th and greater branch orders (*P* < 0.016 in these cases, Bonferroni post-tests; Fig. 3A).

The total dendritic segment surface area per branch order of hypoglossal MNs was dependent on age (F = 13.8, *P* = 0.0003) and branch order (F = 218.6, *P* < 0.0001, 2-Way ANOVA, Fig. 3B). In 24-month-old F344 rats, the total dendritic segment surface area was reduced compared to 6-months-old at the 3rd through the 5th branch orders (*P* < 0.0215 in these cases, Bonferroni post-tests; Fig. 5B).

### Hypoglossal MN dendritic convex hull surface area is reduced in old age

**Fig. 4:**
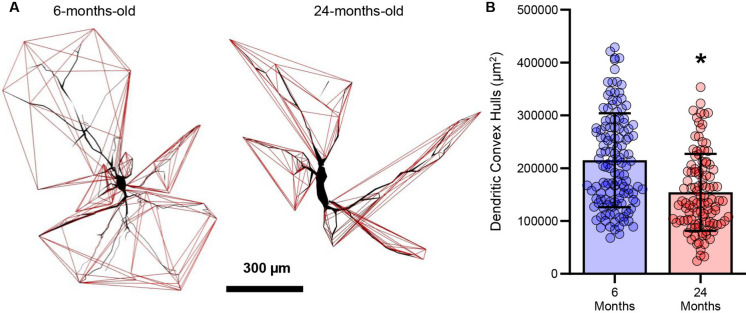
**A **Reconstructed dendritic convex hull surfaces (red polygons) of hypoglossal MNs from 6- and 24-month-old F344 rats. **B **Scatterplot of reduced total dendritic convex hull surface areas of hypoglossal MNs in 24- compared to 6-month-old rats (*P* = 0.0001). Each dot represents one MN (the *n*), * indicates signiificant difference (i.e., *P* < 0.05)

By contrast to the evaluation of passive MN properties derived from length and surface area analyses, convex hull evaluation quantifies the overall receptive field for neural inputs (synaptic and dendro-dendro neuronal contacts) [47]. Here, we assessed the dendritic convex hull surface areas of hypoglossal MNs from 6- and 24-months-old in F344 rats (Fig. 4A). The total dendritic convex hull surface area of hypoglossal MNs was reduced by 28% (*P* < 0.0001, Mann–Whitney U-test; medium effect size d = 0.75) in 24- (154,244 ± 72,978 µm^2^) compared to 6-month-old (214,990 ± 88,905 µm^2^) F344 rats (Fig. 4B).

### Hypoglossal MN dendritic spines are reduced in aging

**Fig. 5:**
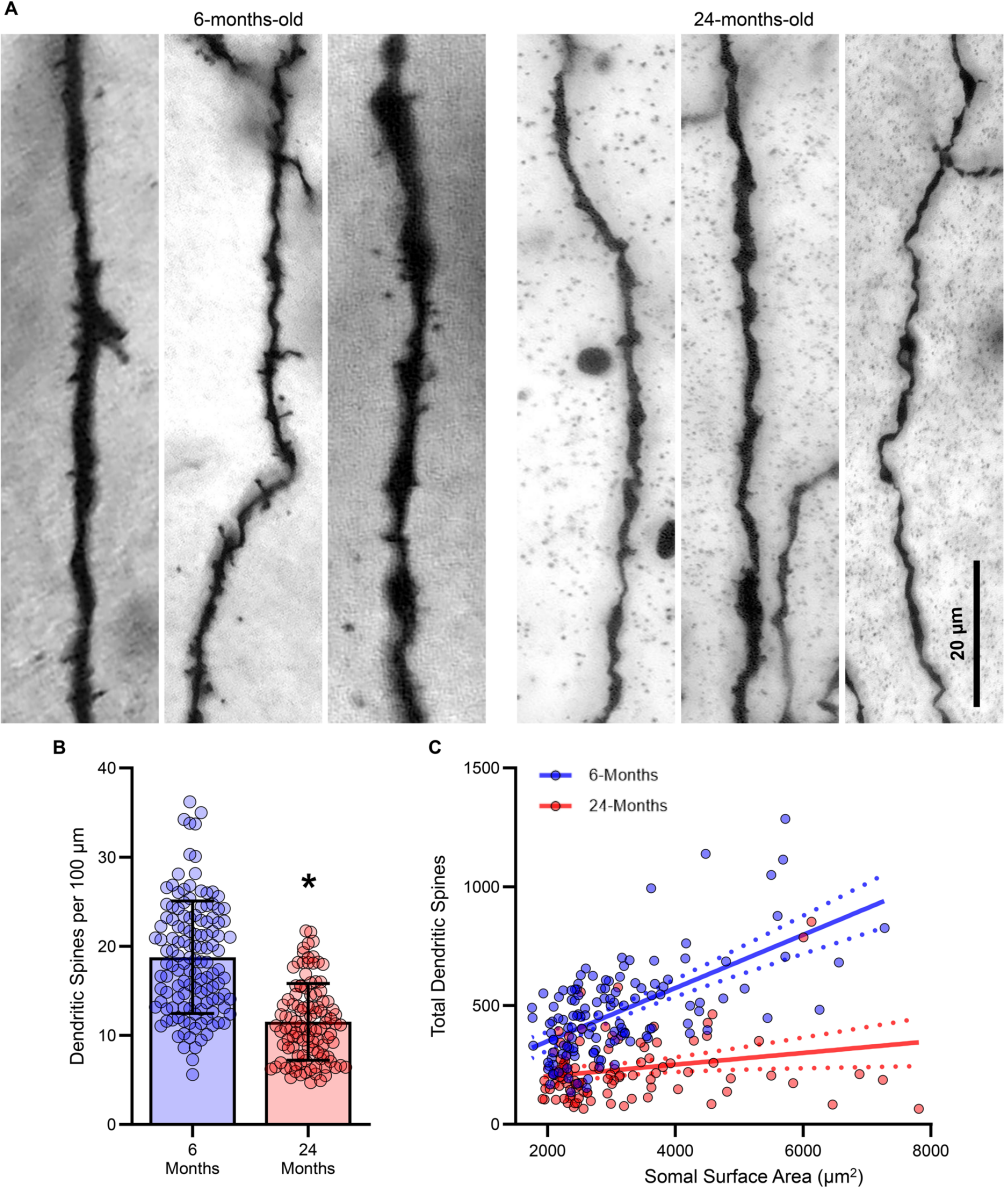
**A **Example micrographs of dendritic spines of hypoglossal MNs from single z-slice images of 6- and 24-month old F344 rats. **B **Scatterplot of reduced dendritic spine density of hypoglossl MNs in 24- compared to 6-month-old rats (*P* = 0.0001). **C **XY plot of the positive linear relationship between hypoglossal MN somal surface area and total dendritic spines in 6- (slope = 0.11, *P* < 0.0001; *r*^2^ = 0.37) and to a lesser extent 24-month-old rats (slope = 0.02, *P* = 0.0216; *r*^2^ = 0.05), with a significant difference between the two co-efficients (*P* < 0.0001). Each dot represents one MN (the *n*), * indicates signiificant difference (i.e., *P* < 0.05)

As a proxy for glutamatergic inputs in a variety of neurons [48], the relative scarcity of MN dendritic spines [33, 35, 49] is somewhat surprising. Nonetheless, the dendritic spines MNs possess are sensitive to altered synaptic inputs [31, 32]. Moreover, in other brainstem MNs, dendritic spine loss in aging precedes MN death [19]. We assessed the dendritic spine density (spines per 100 µm) and the overall number of dendritic spines of hypoglossal MNs from 6- and 24-month-old F344 rats (Fig. 5A). The dendritic spine density of hypoglossal MNs was reduced by 39% (*P* < 0.0001, Mann–Whitney U-test; large effect size d = 1.35) in 24- (11.5 ± 4.3) compared to 6-month-old (18.77 ± 6.306) F344 rats (Fig. 5B). We did not observe any effects of sex on dendritic spine density (Table 1).

The total number of dendritic spines of hypoglossal MNs was reduced by 51% (*P* < 0.0001, *P* < 0.0001, Mann–Whitney U-test; large effect size d = 1.41) in 24- (230.6 ± 135) compared to 6-month-old (470 ± 198) F344 rats.

When we evaluated total dendritic spines with regard to somal surface area, 6-month-old hypoglossal MNs exhibited a linear relationship (slope = 0.11, *P* < 0.0001; *r*^2^ = 0.37), while 24-month-old hypoglossal MN dendritic spines scaled to a lesser extent (slope = 0.02, *P* = 0.0216; *r*^2^ = 0.05; Fig. 5C). The slope of this relationship was reduced ~ fivefold in 24-month-old hypoglossal MNs (*P* < 0.0001; large effect size d = 0.95), indicating larger surface area MNs is where dendritic spine loss was most apparent (Fig. 5C).

### Reduced dendritic mitochondrial volume density in old hypoglossal MN dendrites

**Fig. 6:**
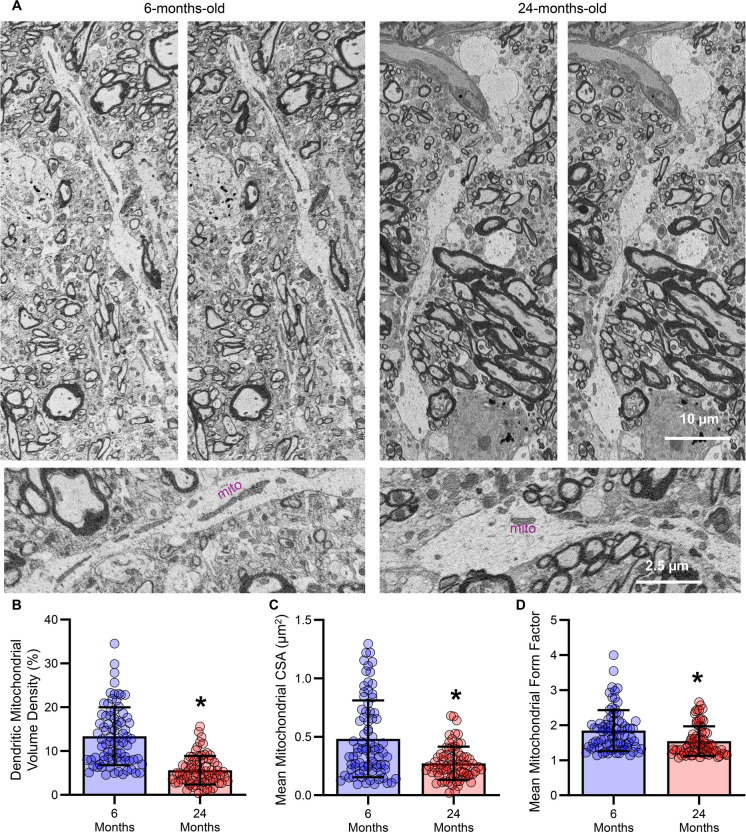
**A **Example serial micrographs of hypoglossal nucleus from 6- and 24-month-old rats, with individual mitochodria easily identified within the dendritic shaft in the larger insets. **B **Scatterplot of reduced dendritic mitochondrial volume density in 24- compared to 6-month-old rats (*P* = 0.0001). **C **Scatterplot of reduced mean dendritic mitochondrial CSA in 24- compared to 6-month-old rats (*P* = 0.0013). **D **Scatterplot of reduced mean dendritic mitochondrial form factor in 24- compared to 6-month-old rats (*P* = 0.0003). Each dot represents one dendrite (the *n*), * indicates signiificant difference (i.e., *P* < 0.05)

**Fig. 7:**
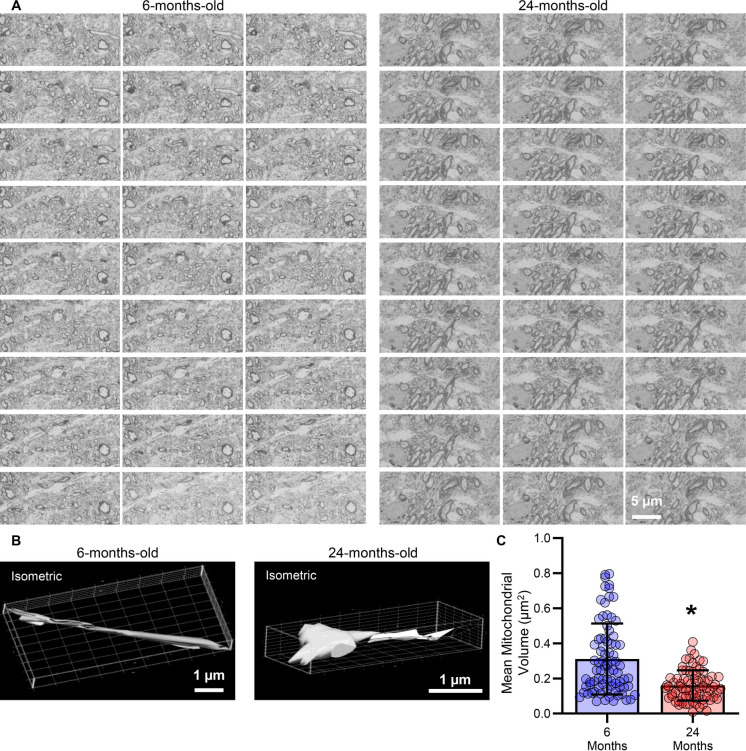
**A **Example serial micrographs (each 75 nm) of hypoglossal MN dendrites nucleus from 6- and 24-month-old rats, with individual mitochodria easily identified within the dendritic shaft. **B **Example isometic projections of a mitonchondrion volume. **C **Scatterplot of reduced dendritic mean mitochondrial volumes in 24- compared to 6-month-old rats (*P* = 0.0001). Each dot represents one dendrite (the *n*), * indicates signiificant difference (i.e., *P* < 0.05)

We evaluated dendrites (6-months-old: *n* = 80; 24-months-old: *n* = 74) within the hypoglossal nucleus for mitochondrial ultrastructure using SBEM [37, 50, 51] (Figs. 6 & 7). The dendritic mitochondrial volume density within the hypoglossal nucleus was reduced by 58% (*P* = 0.0001, Mann–Whitney U-test; large effect size d = 1.49) in 24- (5.61 ± 3.27%) compared to 6-month-old (13.38 ± 6.62%) F344 rats (Fig. 6B). We did not observe any effects of sex on dendritic length or dendritic surface area mitochondrial volume density (Table 1).

### Increased dendritic mitochondrial fragmentation in old hypoglossal MN dendrites

Mitochondrial fragmentation is a common pathologic feature in neurodegeneration [45, 52], commonly assessed using mitochondrial cross-sectional area (CSA) and form factor [22, 45]. We evaluated the CSA and form factor of dendritic mitochondrions within the hypoglossal motor nucleus (Fig. 6). The mean dendritic mitochondrion CSA within the hypoglossal nucleus was reduced by 39% (*P* = 0.0013, Mann–Whitney U-test; large effect size d = 0.83) in 24- (0.273 ± 0.141 µm^2^) compared to 6-month-old (0.482 ± 0.329 µm^2^) F344 rats (Fig. 6C). The mean dendritic mitochondrion form factor within the hypoglossal nucleus was reduced by 16% (*P* = 0.0030, Mann–Whitney U-test; medium effect size d = 0.58) in 24- (1.547 ± 0.422) compared to 6-month-old (1.844 ± 0.584) F344 rats (Fig. 6D). We did not observe any effects of sex on dendritic mitochondrial CSA or form factor (Table 1).

Example mitochondrion volumes reconstructed from SBFEM material shows reduced volume of aged compared to young mitochondria (Fig. 7A & B). Mitochondrial volumes were calculated, with mean mitochondrion volumes reduced by 49% (*P* = 0.0001, Mann–Whitney U-test; large effect size d = 0.97) in 24-(0.160 ± 0.086 µm^3^) compared to 6-month-old (0.311 ± 0.203 µm^3^) hypoglossal MN dendrites (Fig. 7C).

## Discussion

Our findings represent major advances in identifying the motor neuronal degenerative aspects that may contribute to sarcopenia more generally and aerodigestive dysfunctions more particularly. In this study, we found that dendritic structural and mitochondrial changes, consistent with neurodegeneration, were found in old age hypoglossal MNs. Larger (likely type FF) hypoglossal MNs were particularly vulnerable, in agreement with the selective demise of type FF motor units observed in the hypoglossal and other motor pools in old age. These results suggest that synapse loss, as indicated by reduced dendritic spine density and dendritic arbourisation and mitochondrial fragmentation are robust phenotypic markers of degenerating and vulnerable MNs. Our findings are congruent with the altered timing of aerodigestive behaviours in aged F344 rats [19], analogous to dysphagia in elderly humans [53]. The disproportionate dendritic morphology changes in larger hypoglossal MNs are consistent with the motor unit type-specific old age pathophysiology previously observed in F344 rat brainstem and tongue [10, 11], and other respiratory MN pools and muscles [12–14, 23–25, 54]. In our model, 50% survival occurs at 24-months-old, equivalent to ~ 75–80 years old in humans [27, 55]. Notably, elderly humans [44, 56–58] and F344 rats [11, 12, 59–61] exhibit MN death, a phenomenon not observed in the hypoglossal motor nucleus of some other rat strains, nor in mice [62–64]. Thus, we contend that the neurogenic contribution of MNs to the sarcopenia phenotype may be more pronounced in humans and F344 rats than mice.

Neural contributions to altered motor behaviours extend beyond denervation-derived weakness, with diminished neural processing leading to disordered synchronization of muscle activity, a major problem in the pre-frailty population [6, 20, 65]. For example, tongue muscle activations during a variety of aerodigestive behaviours must be synchronised with both the ventilatory cycle and many other bulbar, thoracic and abdominal muscles. This discord likely underpin dysphagia in F344 rats [19]. In nucleus ambiguus neurons of F344 rats, dendritic regression occurs prior to MN death. Here, we show that the neural substrate for hypoglossal MN computation, the dendrites, degenerates in old age, analogous to findings in ALS [33, 66–68]. Hypoglossal MN dendritic spines, although less prevalent than in other neuronal populations [69], are also lost in aged F344 rats. Thus, our dendritic results add to an emerging body of work uncovering the pathophysiology of disordered brainstem neuromotor control and respiratory muscle sarcopenia in aging [4–6, 9–11, 16, 19, 65, 70]. In a major advance, we show that degenerative pathology is predominantly found in the larger hypoglossal MNs (likely type FF units), selectively prone to demise in aging and ALS [11, 71–73]. Our results are consistent with the body of evidence showing that dendritic regression and dendric spine loss are sensitive and specific pathological hallmarks of MN death in MN diseases (ALS) and in old age [19, 21]. These results suggest that strategies preserving the dendritic and dendritic spine cytoskeleton (e.g. pegylated benzothiazoles and strathmin-2) may prove useful in staving off aging neuromotor deficits [43, 74–76].

In general, respiratory MNs and muscles are highly active due to the incessant requirement for ventilation [15]. The high activity of respiratory MNs, and MNs more generally may predispose them to mitochondrial dysfunction during neurodegenerative conditions [77]. Different neuronal compartments have differing requirements for ATP and thus mitochondria [78–81]. Historically the perinuclear region and the axon initial segment, located in the soma, was the focus of most attention, with mitochondria aggregated here to provide for protein homeostasis and action potential generation, respectively [77]. In phrenic MNs, the mitochondrial volume density seems to be inversely correlated with somal surface area [82], indicating that the easily recruited highly active MNs that discharge most readily have more mitochondria to support ATP demand, consistent with modelling approaches [77]. By contrast, larger phrenic MNs, with lower somal mitochondrial volume densities, have a higher abundance of mitochondria within the dendrites [82]. It was assumed in that study that the dendritic arbours of these larger phrenic MNs would be larger [34, 83], and repolarizing the membrane post dendritic excitatory post-synaptic potentials would require more energy in across a larger dendritic surface area (capacitance) [77].

In this study we directly measured the dendritic arbours of smaller and larger MNs, showing directly that somal surface area correlated with dendritic surface area. Notably, in MNs, post synaptic densities do not necessarily act as focal points for mitochondria, as many (perhaps most) MN synapses are directly on the shafts. Thus, we evaluated hypoglossal MN dendritic mitochondrial volume densities in dendritic shafts, with the young control conditions showing mitochondrial volume densities commensurate with the previous (very limited) evaluations in MN dendrites [82]. In a major advance, we show that mitochondrial volume densities are reduced in old age, similar to declines found in hippocampus neurons [81] and striated muscle with age [22]. More importantly, we demonstrate marked mitochondrial fragmentation (reduced mitochondrion CSA, volume and form factors) in aged hypoglossal MNs, similar to age-related changes in mouse hippocampul neurons [81]. It must be noted that mitochondrial fragmentation (fission) is not necessarily pathological, and molecular pathways direct the specific fate of mitochondrions [84, 85]. Indeed, mitochondrial biogenesis relies on mitochondrial fragmentation largely in the central area of mitochondria, while fragmentation at peripheral sites seems to promote mitophagy and degradation [84].

The process of mitochondrial fragmentation is indicative of inflammaging and is heavily implicated in neurodegenerative disorders [45, 52, 86–88] and aging neurons [81]. Mitochondrial morphology is determined by the interaction between two dynamic processes, mitochondrial fragmentation (fission) and mitochondrial fusion [50, 80, 89, 90]. Mitochondrial fission is governed by the abundance of dynamin related protein 1 (DRP1), specifically the phosphorylated form at serine 616 residue [91], and the specific receptor subtypes that bind cytosolic DRP1 to the outer mitochondrial membrane, mitochondrial fission 1 (FIS1), mitochondrial fission factor (MFF), mitochondrial dynamics 49 (Mid49) and mitochondrial dynamics 51 (Mid51) [92–94]. FIS1 receptors largely modulate mitophagy, fission and mitochondrial homeostasis and are heavily implicated in neurodegeneration [93, 94]. The primary receptor related to mitochondrial fission is MFF, as evidenced by over- and under-expression studies [94]. By contrast, Mid49 and Mid51 are closely associated with ER-mitochondrial contact sites and sequesters an inactive form of DRP1 [93, 94]. Sadly, specific DRP1-MFF interaction in aging MNs are currently unknown, although increased DRP1 and/or MFF receptor would be consistent with the current study.

The signalling milieu for mitochondrial fusion also impacts mitochondrial morphology, with mitofusin 2 (MFN2) binding mitochondria to the ER (tethering) and individual mitochondrions to each other [50, 89, 95]. The relative expression levels of DRP1 and MFN2 proteins have been shown to promote mitochondrial fission and fusion, respectively [78, 84, 89, 90]. In striated muscle, reduced MFN2 synthesis with advanced age has been shown to contribute to accumulation of damaged mitochondria and impaired muscle function [96]. The molecular underpinnings of mitochondrial fission and fusion dynamics are poorly (if at all) categorized in MNs of any age, let alone dendrites. Future studies delineating the molecular mechanisms of this MN pathophysiology are of active interest.

Our study has some limitations, the major one being the lack of direct labelling of specific hypoglossal MNs innervating specific tongue muscles (cf. [97, 98]). However, within the hypoglossal nucleus, the majority (> 90%) of the neurons within are MNs [99], and both the golgi technique and SBFSEM approaches are largely incompatible with intracellular fluorescence, given the heavy metal impregnation and monochromatic imaging, respectively. Despite our past success with retrograde fluorescent labelling of adult MNs, the major focus of this paper were the dendritic arbours and spines, which are not resolved adequately, beyond the 2nd – 3rd order dendrites with CTB approaches [82]. As the major pathology was in the distal portion of the dendrites, the drawbacks were considered too great a constraint for retrograde labelling to be considered. In the past, we have successfully electroporated hypoglossal MNs, including extensive evaluation of the dendrites, however, this technique is not viable beyond ~ 1 month postnatal [35, 100], precluding our use of 6- and 24-month-old rats. Likewise, unambiguous evaluation of hypoglossal MN mitochondria and some of the proximal dendritic arbour would be feasible using intravital dye approaches in conjunction with retrograde MN labelling, however, the fine structure of the mitochondria (< 150 nm) is simply not visible and mitochondrial fragmentation evaluations would be highly subjective. In general, although individual tongue muscle motor units may exhibit different populations of resilient (types S and FR) and vulnerable (type FF) motor units [97, 101], our generalized hypoglossal MN dendritic findings are consistent with MN loss in all anatomical regions of the hypoglossal nucleus [11].

At first glance, one may be puzzled by the evaluations of distinct sub-cellular elements, the dendrites spines and mitochondria, beyond the novelty of quantifications being performed in MN dendrites. There are multiple reasons to consider MN dendrites and dendritic spines, and the mitochondria within the dendrites as being highly physiologically related to one another. First, mitochondria are concentrated at central synapses in the pre- and postsynaptic regions of synapses, particularly dendritic spines [80, 81, 102]. Indeed, fragmentation of presynaptic mitochondria, such as those observed here, diminish synaptic function [103]. Impaired synaptic mitochondrial function may plausibly underlie early circuit deficits observed in F344 neuromotor behaviour [19]. Secondly, maintaining the polarized state of the dendritic membrane requires greater mitochondrial abundance and ATP production than even the axon compartment [81, 104]. In this study and in prior studies of brainstem and spinal MNs, it has been shown that the type FF MNs (that are vulnerable to aging) have larger dendritic lengths and surface areas compared to type S or FR units [34, 67, 105, 106]. A greater capacitance of these FF motor units requires greater action of ATP to restore membrane potential after a local depolarization. Although not directly tested, this increased burden on type FF dendritic mitochondria may serve as the physiological basis for the vulnerability of these MNs to degeneration and demise. The third clue as to the relationship linking synapse loss and mitochondrial fragmentation is from the most common familial mutant of ALS, SOD1. Various SOD1 mutant approaches produce MN mitochondrial fragmentation and dysfunction [26, 45, 107] and, in addition, produce spine and/or synapse loss [33, 108]. The timeline and which direction (if any) causality occurs in the relationship between synaptic and mitochondrial defects in aging MNs is a key discovery if we are to fully understand neuromotor aging.

In conclusion, we present compelling dendritic data, showing that synapses and mitochondria within the dendrites are highly compromised at old age. Although synapse loss and mitochondrial breakdown are known harbingers of neuronal death [26, 45, 87, 88, 109], the major pathophysiological contribution of dendritic abnormalities in age may be altered neuromotor processing [20]. These processing alterations are particularly problematic in the respiratory system, where any breakdown in the coordination of aerodigestive behaviours and inspiration predisposes to aspiration. We propose that future work aimed at alleviating synapse loss and mitochondrial breakdown in aged MNs may improve aerodigestive behavioural function in addition to preserving MNs staving off sarcopenia [20]. These approaches may prove to be of great utility in human populations at risk of frailty, including those suffering dementia, OSA, or undergoing chemotherapy [110–112].

## Data Availability

All of the data are presented individually in the results, with original data available upon request form the corresponding author.
